# Sensitivity of larval and juvenile fish with different swim bladder morphology to barotrauma with a special focus on Cypriniformes

**DOI:** 10.1038/s41598-025-32670-y

**Published:** 2026-01-07

**Authors:** Andreas Zitek, Wolfgang Gessl, Peter Mehlmauer, Clemens Ratschan, Martin Schletterer, Josef Schneider

**Affiliations:** 1 EcoScience, Vienna, 1140 Austria; 2 Institute of Biology, University of Graz, Graz, 8010 Austria; 3 Self-employed, Graz, 8020 Austria; 4 Consulting Office for Applied Hydrobiology, Fisheries and Hydro-Engineering, TB Zauner GmbH, Engelhartszell, 4090 Austria; 5 Department of Hydropower Engineering, TIWAG -Tiroler Wasserkraft AG, Innsbruck, 6020 Austria; 6 Institute of Hydrobiology and Aquatic Ecosystem Management, Department of Ecosystem Management, Climate and Biodiversity, BOKU University, Vienna, 1180 Austria; 7 Institute of Hydraulic Engineering and Water Resources Management, Graz University of Technology, Graz, 8010 Austria

**Keywords:** Barotrauma, Swim-bladder morphology, Cypriniformes, Characiformes, Kaplan-turbine, Downstream migration, Ecology, Ecology, Ocean sciences

## Abstract

Barotrauma, caused by rapid pressure changes, poses a major risk to fish migrating downstream through hydropower turbines. This study investigated the sensitivity of larval and juvenile fish with different swim bladder morphology to barotrauma using a custom-built chamber. Four different representative species, namely the two cypriniform species common nase (*Chondrostoma nasus*) and roach (*Rutilus rutilus*), both physostomous species with a two-chambered swim bladder, European grayling (*Thymallus thymallus*) as physostomous species with a single-chambered swim bladder and European perch (*Perca fluviatilis*) as physoclistous species with a single chambered swim bladder were investigated. Fish were acclimated to 0- or 15-meters depth (101 and 251 kPa, respectively) and exposed to rapid decompression to different nadirs (15, 30, 40, 60 kPa) to simulate turbine passage as a basis to construct dose-response curves predicting lethal injury probabilities. Species- and stage-specific injury patterns emerged, with physostomous cypriniforms showing the highest sensitivity and suffering frequent swim bladder ruptures. Depth acclimation, particularly in E. grayling, increased vulnerability. Moreover, lower barotrauma-related mortality was observed under partial load conditions in Kaplan turbines compared to full load. These insights are of high relevance for predicting barotrauma-related injury risks across species, particularly for species-rich groups like Cypriniformes and Characiformes that share similar swim bladder traits, to support sustainable hydropower development.

**Supplementary Information:**

The online version contains supplementary material available at 10.1038/s41598-025-32670-y.

## Introduction

Hydroelectric power plants are present in rivers worldwide, providing renewable energy^1^ but also posing significant risks to aquatic life e.g. by habitat degradation, habitat fragmentation and disruption of migration routes^2,3^ as well as by potentially causing injuries during turbine passage^4,5^.

Injuries and mortality of fish passing through turbines of hydroelectric power plants can result from rapid and extreme pressure changes, shear stress, turbulence, collision with structures (“strike”) or grinding^5^, including delayed mortalities after passing through a turbine^6–8^ and cumulative effects due to multiple turbine passages^9,10^. Moreover, an increased risk of predation after turbine passage may constitute an additional source of fish mortality^11–13^.

Mortalities caused by turbine passage therefore might lead to recognizable negative effects on population level^14,15^. Out of the described types of injuries, the most relevant physical sources of mortal injuries for fish during turbine passage are considered to be direct contact with parts of the turbines such as blades (“blade strike”)^16–19^ as well as sudden pressure changes (so-called “barotrauma”)^20–24^, with minor effects from shear forces and strong turbulence^25–27^.

Barotrauma injuries are primarily caused by the rapid expansion of the swim bladder when fish experience sudden decompression, usually within a fraction of a second^20,28^, during turbine passage^20^ or the capture and extraction of fish from greater water depths^29^. The ratio of acclimation pressure to nadir pressure (lowest experienced pressure), also called Ratio of Pressure Change (RPC), hereby is decisive in determining the severity of injuries, making the expected barotrauma damage dependent on the migration depth of fish and the associated pressure adjustment required within the swim bladder^23^.

However, it has to be noted that depending on the pathway of fish through the turbine, they might be exposed to different nadir pressures, which can be either determined by Computational Fluid Dynamics (CFD) modelling^22^, or a significant number of data collected by different types of pressure devices^30–33^.

Typical documented injuries caused by barotrauma are ruptured swim bladders, internal bleeding (hemorrhaging), blood or bile exiting from the mouth, exophthalmia (protrusion of eyes and swim bladder), and gas bubbles (emboli) in the vasculature, organs, gills and fins^20,23,34,35^.

Especially the species-specific swim bladder anatomy, developmental stage and the associated status of swim bladder development, as well as behavioral patterns during downstream migration mainly related to potential depth acclimation, are considered as decisive for the differences in susceptibility for injuries related to swim bladder expansion and rupture between species^23^.

Swim bladders have evolved in fish as a means of buoyancy control, although in some species they fulfil auxiliary functions such as respiration, sound production, and hearing^36,37^, and show a high structural diversity in teleostean fish^38^. Generally, based on their swim bladder structure, fishes can be classified into so-called physostomes, physoclists, and species with no swim bladder^39^. In physostomous species of e.g. the orders of Cypriniformes, Characiformes, Clupeiformes, Salmoniformes and Siluriformes the swim bladder is connected to the esophagus via a pneumatic duct^23^ allowing for autonomic gas release due the so called “Gasspuckreflex” (gas spitting reflex)^40–42^. This reflex is being considered to be critical in lowering the likelihood of harm due to rapid pressure changes but being variable between and within species^23^, and related to the already described speed of pressure drop during turbine passage. Physostomes can either have a single-chambered swim bladder or a two-chambered swim bladder^38^.

While Salmoniformes possess only a single-chambered swim bladder, e.g. in Characiformes and Cypriniformes, the swim bladder typically consists of two parts, with only the posterior chamber of the swim bladder directly connected to the esophagus via the pneumatic duct^23,43–45^. Both swim bladder chambers are separated by a muscle that maintains a balanced pressure in the anterior chamber^46^. To equalize pressure in the anterior chamber in the event of a rapid reduction of external pressure, gas needs to pass through the communicating duct between the anterior and posterior chambers before it can be expelled via the pneumatic duct^47^. The anterior part of the swim bladder in cyprinids is structurally reinforced by specific tissues and directly connected to the so-called “Weberian ossicles”, specialized structures associated with hearing^46,48^.

While in physoclists the swim bladder is open only during a very early stage during ontogeny for an initial filling and degenerates later^49,50^, in physostomous species the pneumatic duct is maintained^50^. While in physostomous species like salmonids the whole swim bladder is filled by ingesting air through the pneumatic duct after the yolk sac is absorbed^51^, cyprinids first fill the posterior chamber^52^. The anterior chamber of the swim bladder is filled later during ontogeny^53^.

Previous barotrauma studies have primarily focused on salmonid species being physostomes with a single-chambered swim bladder^20,22,34,54,55^, or perciform species of the physoclistous group^56–58^, with physoclistous species being considered as much more susceptible to barotrauma injuries related to swim bladder rupture than physostomous species^23^. Barotrauma-related mortalities of up to 100% for juvenile European percids were documented by Barus et al.^59^ and Barus et al.^60^ after turbine passage. Only a few studies documented severe effects of barotrauma on swim bladders in cyprinids with a two-chambered swim bladder^61,62^ or neotropical physostomous species with similar swim bladder morphology^35^. Knowledge on vulnerability to barotrauma is therefore mostly available for selected salmonid and percid species^55,58^, and for individual other species like the American sturgeon *Acipenser transmontanus*^63^, the lamprey *Lampetra richardonii*^64^, the Amazonian catfish *Pimelodus pictus*^65^, one typical cyprinid species, the crucian carp *Carassius carassius*^62^ and two neotropical fish species (*Piaractus mesopotamicus* and *Brycon orbignyanus*) with a two-chambered swim bladder^35^.

Despite the known differences in swim bladder morphology in different species and life stages, comparative studies investigating differences in barotrauma injuries between species are lacking. Given the extensive global expansion of hydropower projects in Neotropical^35^ and other tropical regions^66–69^, where many species possess a multi-chambered swim bladder architecture, there is an urgent need to better understand how species and life stages with different swim bladder architecture are affected by rapid pressure changes during turbine passage.

On a global level, Lévêque et al.^70^ reported 12,740 freshwater fish species with 350 species occurring in Europe, while South America and Asia host 4,035 and 3,553 freshwater fish species, respectively. The dominant orders are Cypriniformes (3,451 species), Siluriformes (2,835), Perciformes (2,402 species) and Characiformes (1,794 species). In contrast, the order of Salmoniformes only contains 161 species^70^. However, the total number of fish species worldwide and of those living constantly in freshwater habitats is continuously being updated. According to Eschmeyer’s Catalog of Fishes, a total of 37,209 fish species are currently recognized world-wide, of which 18,962 inhabit freshwater^71^. Many of the latter species may be affected by run-of-river hydropower plants (RoR-HPPs) during downstream migration. However, it remains unclear to what extent barotrauma-related injuries may affect different species of the dominant order of Cypriniformes during downstream migration, given that they are physostomes with a two-chambered swim bladder.

This is the reason, why this study focused on the systematic and comparative evaluation of barotrauma effects on larval and juvenile young-of-the-year stages of selected typical European river fish species of global relevance with different swim bladder morphology during simulated turbine passage using a custom-designed barotrauma chamber^72^. In addition, due to animal ethics requirements, potential effects of anesthesia on results also were assessed in this study.

## Results

### Effect of aesthesia (MS222)

Experiments indicated no significant difference in mortal injuries between anesthetized and non-anesthetized fish for all species and life stages tested (E. grayling L1/L2, 0+, E. perch L1/L2, L4, 0+, roach 0 + and E. perch L4 which were only available for pre-experiments) as determined by Fisher’s exact and Chi-square tests (**Supplementary Tab**). This was confirmed by regression analyses (L4 perch B = -0.149, *p* = 0.764, OR = 0.862; 0 + perch B = 0.128, *p* = 0.698, OR = 1.137; 0 + roach B = -0.227, *p* = 0.497, OR = 0.797; no useful models could be determined for L1/L2 grayling, L 1/L2 perch and 0 + grayling due to the low mortality rates).

## Documented sensitivity to changes - types of injuries

The most abundant type of injuries at all studied species and life stages, except for L1/L2 larvae of E. grayling and E. perch who are lacking a swim bladder at this stage, were ruptured swim bladders, often associated with intraperitoneal gas bubbles (in 0 + individuals of roach, nase, E. grayling and E. perch), a ruptured abdominal wall (E. perch) or ruptured peritoneum (nase) (Fig. 3; Table 4). In 0 + and L6/J1 cyprinids the anterior swim bladder was mostly subjected to rupture, while in early L1 nase only the posterior part existing during this stage was ruptured.

**Fig. 1:**
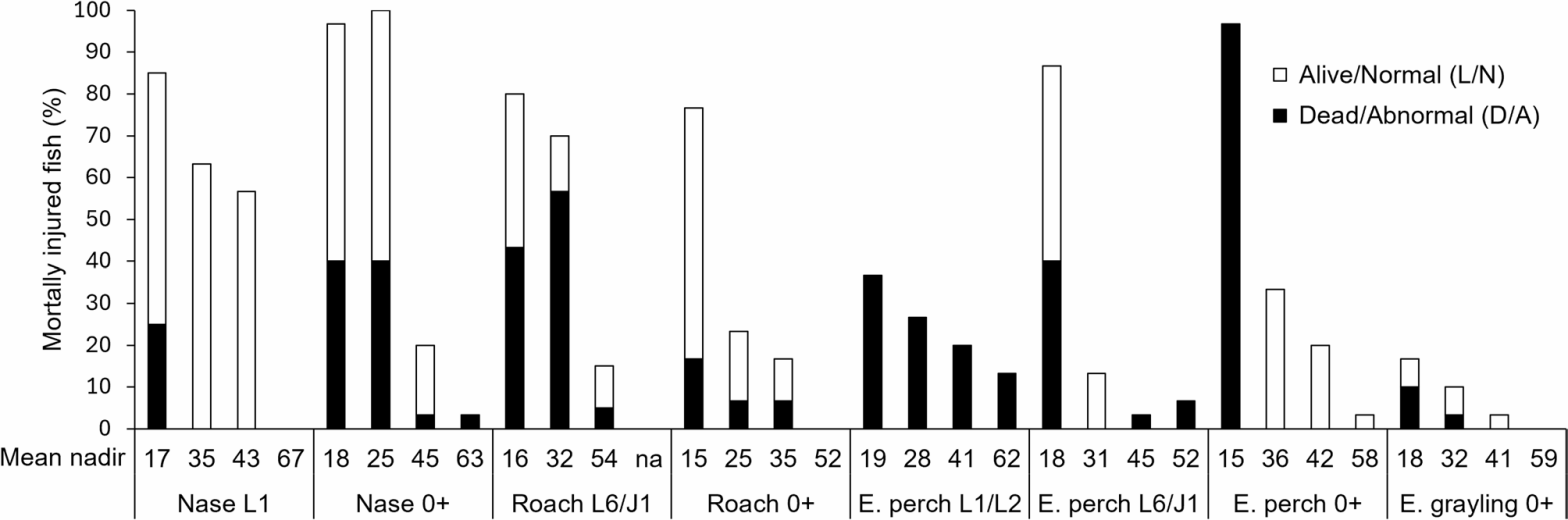
Percentage of mortally injured fish divided into dead/abnormal (D/A) and alive/normal (L/N) after 24 h per studied species/life stage at different nadirs (mean values of nadirs of all trials are given in kPa); find more detailed information in Table 1.

As Table 4 (in the method section) does not allow for a direct comparison of the documented injuries between species, as not all of them were subjected to the same treatments (pre-experiments, atmospheric pressure experiments, acclimation experiments), Table 1 presents the comparable results of the atmospheric pressure trials used to create dose response curves with a focus on swim bladder ruptures. An overall finding was that especially in cyprinids like L1 and 0 + of nase and 0 + of roach, but also in L6/J1 E. perch, a large proportion of the fish were still alive and not behaving abnormally after 24 h although suffering from ruptured swim bladders in most cases, which is highlighted in Fig. 1.

In L1 nase (*n* = 130) subjected to systematic pressure drops, ruptured swim bladders and internal bleeding were identified as fatal injuries (Table 4). Only 10 individuals (7.7%) were dead/abnormal after 24 h, with a majority of fish still being alive after 24 h. But 58 (38.5%) of these had ruptured swim bladders, with 10 of them also suffering from internal bleeding. Two individuals were classified as fatally injured only due to internal bleeding. This yielded a total of *n* = 70 (53.8%) mortally injured fish.

In L1/L2 E. perch, due to the difficulty of clearly assessing injuries, only dead and abnormally behaving individuals were classified as fatally injured. Due to the early developmental stage, only a few of them had already a developed swim bladder.

For the L6/J1 E. perch, in addition to the dead and abnormal individuals (*n* = 15, 12.5%), those with a ruptured swim bladder or where internal bleeding was visible were additionally classified as mortally injured (*n* = 18, 15%). This yielded a total of *n* = 33 (27.5%) mortally injured fish. Since mortally injured individuals were recorded in both the control and experiments, the mean (in this case, 1 individual) of the control mortality was corrected for.

In the L6/J1 stage of roach, in total 31 individuals (38.8%) were dead/abnormal after 24 h, while additionally 17 individuals (21.3%) with ruptured anterior swim bladders (*n* = 14, out of which *n* = 11 had intraperitoneal gas bubbles) and, two with ruptured posterior swim bladders (both associated with intraperitoneal gas bubbles and one associated with hemorrhage at the posterior swim bladder), were used as markers for mortal injury. This yielded a total of *n* = 48 (60%) of mortally injured fish. Due to the small number of fish available, the experiments with nadir 60 kPa were omitted, and only two replicates were conducted at nadir 40 kPa and control.

In 0 + roach, *n* = 9 individuals (7.5%) were dead/abnormal after 24 h with 26 living/normal individuals (21.7%) classified as mortally injured after 24 h. 25 of them had a ruptured anterior swim bladder and intraperitoneal gas bubbles. This yielded a total of *n* = 35 (29.2%) mortally injured fish. Lethal injuries documented were ruptured anterior swim bladders, intraperitoneal gas bubbles, gas bubbles in the blood vessels, and ruptured abdominal walls.

In 0 + nase 26 individuals (21.7%) were dead/abnormal after 24 h. Those individuals still alive/normal after 24 h were classified as mortally injured mainly due to ruptured anterior swim bladders (*n* = 30), but also due to ruptures of the peritoneum (*n* = 10). This yielded a total of *n* = 66 (55%) mortally injured fish.

In 0 + E. grayling 4 individuals (3.3%) were dead/abnormal after 24 h. In addition, living/normal grayling with ruptured swim bladders (*n* = 5) were classified as mortally injured, with four of these also having intraperitoneal gas bubbles. This yielded a total of *n* = 9 (7.5%) mortally injured fish.

In 0 + E. perch, *n* = 29 individuals (24.2%) were dead/abnormal after 24 h. Sixteen out of seventeen 0 + perch were alive but showed highly abnormal immobility at the cage bottom after 24 h and were classified as mortally injured due to abdominal wall ruptures, with one individual suffering internal/visceral bleeding. This yielded a total of *n* = 46 (38.3%) of mortally injured individuals.

**Table 1:** Percentages of mortally injured (MI) individuals divided in dead/abnormal (D/A) and alive/normal (L/N) fish in relation to the documented percentages ruptured swim bladders from main experiments at target Nadirs of 15, 30, 40 and 60 kPa (3*10 individuals for each target nadir, except for L6/J1 roach at target Nadirs 40 and 60 kPa due to the lack of individuals) with the experimentally achieved nadirs; note that L1/2 E. grayling are not represented as there were no mortally injured individuals noted during atmospheric pressure experiments and only two individuals at pre-experiments.

Species / Life stage	Total number of fish *n*	Real nadirs kPa	Total number of fish %	Total number of fish %		Ruptured anterior swim bladder (Cypr.) %		Ruptured posterior swim bladder (Cypr.) %			Both swim bladder sections ruptured (Cypr.) %		Ruptured swim bladder (one chamber) %	
			MI	D/A	L/N	D/A	L/N	D/A		L/N	D/A	L/N	D/A	L/N
Nase L1	40	16, 16, 17, 19	85.0	25.0	75.0			25.0		60.0				
	30	35, 33, 38	63.3	0	100			0		63.3				
	30	40, 43, 45	56.7	0	100			0		50.0				
	30	62, 68, 71	0	0	100			0		0				
	*30*	*Control*	*0*	*0*	*0*			*0*		*0*				
Nase 0+	30	19, 19, 17	96.7	40.0	60.0	40.0	40.0	10.0		0	10.0	0		
	30	23, 28, 23	100	40.0	60.0	36.7	53.3	0		0	0	0		
	30	44, 45, 45	20.0	3.3	96.7	0	6.7	0		0	0	0		
	30	61, 63, 64	3.3	3.3	96.7	0	0	0		0	0	0		
	*30*	*Control*	*0*	*0*	*100*	*0*	*0*	*0*		*0*	*0*	*0*		
Roach L6/J1	30	16, 18, 15	80.0	43.3	56.5	43.3	33.3	6.7		13.3	6.7	10.0		
	30	31, 33, 32	70.0	56.7	43.3	56.7	6.7	0		6.7	0	0		
	20	48, 59, n.a.	15.0	5.0	95.0	0	10	0		0	0	0		
	0	n.a.	n.a.	n.a.	n.a.	n.a.	n.a.	n.a.		n.a.	n.a.	n.a.		
	*20*	*Control*	*0*	*0*	*100*	*0*	*0*	*0*		*0*	*0*	*0*		
Roach 0+	30	15, 16, 15	76.7	16.7	83.3	13.3	60.0	3.3		0	3.3	0		
	30	25, 28, 23	23.3	6.7	93.3	3.3	16.7	0		0	0	0		
	30	37, 32, 35	16.7	6.7	93.3	3.3	6.7	0		0	0	0		
	30	54, 54, 49	0	0	100	0	0	0		0	0	0		
	*30*	*Control*	*0*	*0*	*100*									
E. perch L1/L2	30	18, 19, 21	36.7	36.7	63.3								3.3	3.3
	30	26, 29, 30	26.7	26.7	73.3								0	0
	30	41, 42, 41	20.0	20.0	80.0								0	6.7
	30	63, 61, 62	13.3	13.3	86.7								0	3.3
	*30*	*Control*	*23.3*	*23.3*	*76.7*								*0*	*0*
E. perch L6/J1	30	19, 17, 18	86.7	40.0	60.0								40.0	46.7
	30	30, 32, 32	13.3	0	100								0	13.3
	30	45, 45, 45	3.3	3.3	96.7								0	0
	30	53, 52, 52	6.7	6.7	93.3								0	0
	*30*	*Control*	*6.7*	*3.3*	*96.7*								*0*	*0*
E. perch 0+	30	15, 15, 15	96.7	96.7	3.3								23.3	0
	30	34, 36, 37	33.3	0	100								0	0
	30	41, 41, 45	20.0	0	100								0	0
	30	62, 59, 54	3.3	0	100								0	0
	*30*	*Control*	*0*	*0*	*100*								*0*	*0*
E. grayling 0+	30	19, 19, 17	16.7	10.0	90.0								6.7	6.7
	30	31, 32, 32	10.0	3.3	96.7								3.3	6.7
	30	41, 39, 43	3.3	0	100								0	3.3
	30	62, 59, 57	0	0	100								0	0
	*30*	*Control*	*0*	*0*	*100*								*0*	*0*

## Prediction of sensitivity to pressure changes

**Fig. 2:**
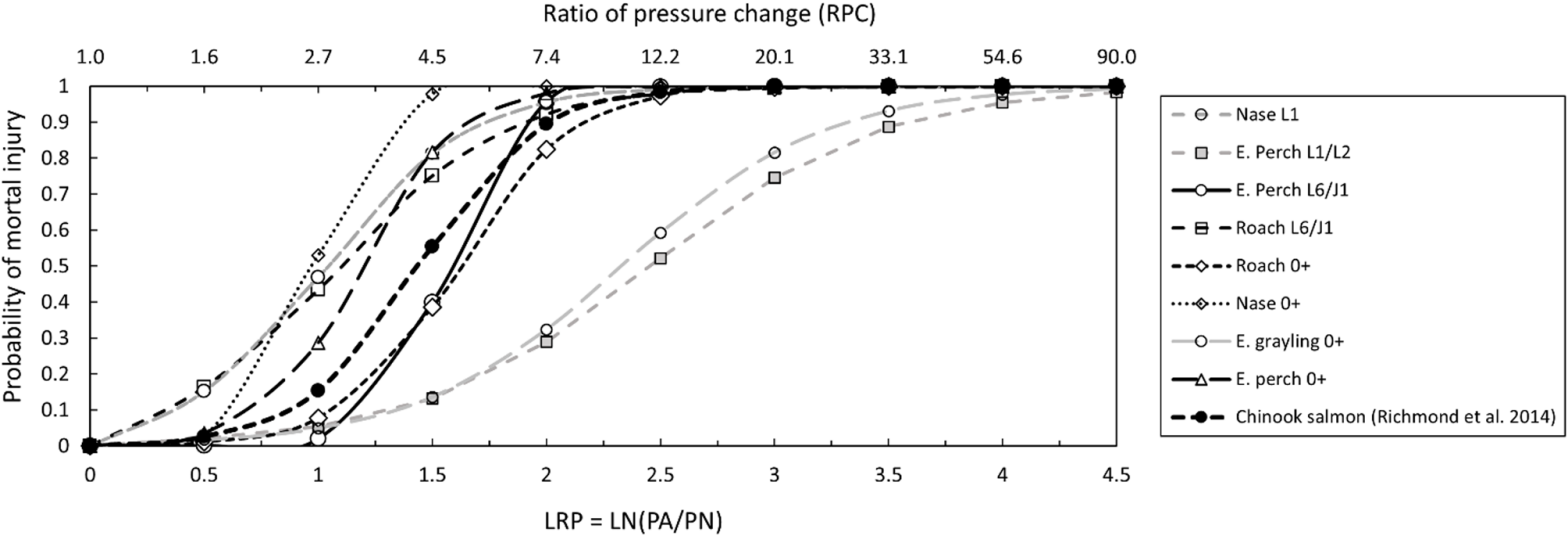
Mortal injury probabilities along a range of LRP (natural log of the ratio of acclimation to nadir pressures, LN(P_A_/P_N_)) and ratios of pressure changes (RPC) for all examined fish species and developmental stages except for L1/L2 E. grayling compared to those reported for Chinook salmon^22^.

The resulting logistic regressions LN(acclimation pressure P_A_/nadir pressure P_N_) clearly indicated species- and life stage-specific mortal injury probabilities (Fig. 2). Nase (L1, 0+) and roach (L6/J1) as physostomous species exhibited higher injury probabilities (≥ 50% mortally injured fish) at an RPC of 2.7 than European perch (L6/J1, 0+). E. grayling 0 + juveniles and early juvenile perch stages without fully developed swim bladders showed the lowest susceptibility with ≥ 50% mortally injured fish at an RPC between 10 and 12. For L1/L2 E. grayling, no injuries occurred, thus, no injury function was derived.

## Depth acclimation experiments

Depth-acclimated 0 + juveniles (15 m depth, 251 kPa) of E. grayling demonstrated heightened sensitivity to pressure changes, even when decompressed only slowly (rate of pressure change ROC = 3.3 kPa s^− 1^) to atmospheric pressure (ratio of pressure change RPC = 2.5) during 45 s. 94% and 90% of 0 + grayling suffered from fatal swim bladder ruptures at rapid decompression from 251 kPa to nadirs of 42 kPa (RPC = 6) and 44 kPa ( RPC = 5.7) respectively, and 47% at a slow pressure change from 251 kPa to atmospheric pressure of 101 kPa (Table 2). 0 + of nase, E. perch, and roach showed expected high mortal injury rates upon depressurization to nadirs of 29 kPa, 15 kPa and 44 kPa respectively, and only nase suffered 9% mortality when slowly returned to atmospheric pressure (RPC = 2.5, ROC = 3.3 kPa s^− 1^).

**Table 2:** Mortal injuries for 0 + individuals of roach, nase, E. perch, and E. grayling acclimated to 251 kPa. For E. grayling, experiments were conducted for both anesthetized and unanesthetized individuals. The table includes nadirs, the rates of pressure change (ROC, kPa s^− 1^) and ratio of pressure change (RPC) values, as well as mortal injury rates predicted using the regression equations from the atmospheric pressure trials, compared with the actual injury rates observed during the experiments.

Species / Life stage	Acclimation pressure (kPa)	Nadir (kPa)	ROC (kPa s^− 1^)	RPC	% mortal injuries calculated	% mortal injuries documented
Roach 0+	251	44	2,070	5.7	62.3	79.0
Roach 0+	251	101	3.3	2.5	5.5	0.0
Nase 0+	251	29	1,480	8.7	100.0	100.0
Nase 0+	251	101	3.3	2.5	36.3	9.0
E. perch 0+	251	15	1,180	16.7	100.0	100.0
E. perch 0+	251	101	3.3	2.5	20.4	0.0
E. grayling 0+ (anesthetized)	251	44	2,070	5.7	21.2	90.0
E. grayling 0+ (not anesthetized)	251	42	1,990	6.0	23.0	94.0
E. grayling 0+	251	101	3.3	2.5	4.0	47.0

## Turbine passage simulations

Particle simulations performed using the Ansys CFX V17.1 software indicated notable differences in nadir distributions between the studied low-head Run-Of-River Hydropower-Plants (RoR-HPPs) and load conditions (**Supplementary Table 2**). Significant differences in mortal injury rates between full and partial load turbine operations were yielded (for the parameters of the turbines and RoR-HPPs see **Supplementary Table 5**). Highest percentages of mortally injured fish during turbine passage were calculated for nase L1 (8.2%), roach L6/J1 (7.8%), nase 0+ (8.0%), E. perch 0+ (5.9%) at full load with reduced injuries occurred at partial load (Table 3).

**Table 3:** Summary of the model parameters *b*0 and *b*1 for the logistic regression, and calculated percentage of mortally injured fish passing through a turbine (percent mortality = % M) based on the application of the logistic regression to the nadir distributions determined by the particle simulations for full load (FL) and partial load (PL) conditions at the two studied low head Run-Of-River Hydropower-Plants (RoR-HPPs) for surface-acclimated fish.

Species/Life stage	Model parameter		RoR-HPP 1				RoR-HPP 2			
	b0	b1	% M FL		% M PL		% M FL		% M PL	
E. grayling L1/L2	No mortal injuries		0		0		0		0	
Nase L1	-3.319	3.194	8.2		0.80		10.7		2.0	
E. perch L1/L2	-4.848	1.974	1.4		0.10		1.9		0.9	
E. perch L6/J1	-10.909	6.998	2.4		0.04		1.9		0.9	
Roach L6/J1	-3.003	2.737	7.8		0.78		10.5		2.0	
Roach 0+	-6.492	4.017	2.8		0.12		2.6		1.0	
Nase 0+	-7.257	7377	8.0		0.56		8.1		1.4	
E. grayling 0+	-5.182	2.222	1.4		0.09		1.9		0.9	
E. perch 0+	-5.720	4.806	5.9		0.36		5.9		1.3	

## Discussion

The present study demonstrates a pronounced species- and stage-specific susceptibility to barotrauma during simulated turbine passage. Particularly high susceptibility and higher injury probabilities were noted for all the investigated stages and species of physostomous Cypriniformes such as common nase (*Chondrostoma nasus*, L1, 0+) and roach (*Rutilus rutilus*, L6/J1), compared to a physoclist species (European perch *Perca fluviatilis*, L6/J1, 0+) and a phyostomous salmonid species (European grayling *Thymallus thymallus*, L1/L2, 0+). For L1 nase and L6/J1 of roach probabilities of mortal injuries started to increase at a ratio of pressure change (RPC) as low as 1.6 reflecting nadir pressures between 60 and 65 kPa. This provides strong empirical support that physostomous species with a two-chambered swim bladder might be even more vulnerable to rapid decompression due to physiological constraints in their capacity to regulate internal gas pressure than physoclist species.

The anatomical and physiological characteristics of the cyprinid swim bladder explain this heightened sensitivity. While physostomous fishes generally possess a pneumatic duct that, in principle, enables the release of gas from the swim bladder into the digestive tract (“gas-spitting reflex”^40–42^, in Cypriniformes the pneumatic duct connects only the posterior chamber to the esophagus. The anterior chamber - structurally reinforced and connected with the Weberian apparatus - can vent gas only indirectly through a narrow connecting duct leading to the posterior chamber^47^. During rapid decompression, this limited pathway seems to be insufficient for fast gas release, which leads to the frequent rupture of the anterior part of the bladder. The high incidence of anterior-chamber ruptures in nase and roach matches findings from European and neotropical physostomous species with similar swim bladder morphology^35,62^. Together, these results highlight that the combination of a two-chambered structure and a restricted release pathway constitute a major intrinsic risk factor for barotrauma in these groups.

Ontogenetic stage further modulates barotrauma sensitivity. Individuals at early larval stages of perch and grayling (L1/L2), which do not yet possess a developed swim bladder, were the least vulnerable, as there is no gas-filled organ available to be expanded during decompression. In perch, the bladder inflates only near the end of the L2 stage^73^ and in grayling at the L3 stage^74^. Before these developmental steps, fat droplets serve as the hydrostatic organ. In contrast, early cyprinid larvae, such as nase L1, already possess a partially developed posterior swim bladder whose thin wall and fragile tissues are easily damaged^52,53^. Even without prior depth acclimation, moderate pressure decreases can cause ruptures once gas is present, especially if the pneumatic duct is not yet fully functional. Thus, morphological fragility, gas dynamics and the developmental timing of swim bladder inflation jointly influence the vulnerability of early life stages.

Across all experiments involving pressure reduction, ruptured swim bladders were by far the most common injury wherever a bladder was present. Ruptured swim bladders were often accompanied by intraperitoneal gas bubbles, ruptured abdominal walls (0 + E. perch) or ruptured peritonea (0 + nase). Internal bleeding (L1 nase, L6/J1 perch, 0 + E. perch) and eye hemorrhaging (0 + nase and 0 + roach) were also significant injuries. In cyprinids in particular, rupture of the anterior chamber was the dominant injury type, in agreement with findings from barotrauma studies studying comparable species^35,61,62^.

Notably, many fish appeared externally normal despite severe internal damage, which aligns with earlier observations that delayed mortality can be substantial^56,61,75^. Mueller et al.^75^ documented delayed mortality increasing after 96 h especially when intraperitoneal gas was present. In crucian carp with high rates of swim bladder rupture after rapid decompression also external signs of trauma became more pronounced over time^62^. Mueller et al.^75^ found a high prevalence of internal gas, bladder anomalies, and vertebral fractures in E. grayling and Danube salmon (*Hucho hucho*) after experimental turbine passage. Because such injuries can compromise buoyancy, feeding, swimming performance, and predator avoidance, indirect mortality is likely high even if fish survive initial trauma^57^. Given this complexity, the use of a “mortal injury metric”^76^ is essential to estimate delayed mortality, although such metrics might still underestimate indirect ecological consequences.

A central finding of this study is the increase of injury probabilities especially for physostomous cyprinid species already at relatively low ratios of pressure change (RPCs). For surface-acclimated nase larvae, nase 0+, and roach L6/J1, mortal injury probabilities increased at RPC values as low as 1.6 (nadirs between 60 and 65 kPa) and exceeded 50% at RPC 2.7 (nadir around 37 kPa). By contrast, juvenile Chinook salmon showed comparable mortality at an RPC of approximately 4^34^. Across species examined here, RPCs in the range of 2.7-5 consistently produced at least 50% mortality in surface-acclimated fish, excluding early-stage perch and grayling. Neotropical characids with two-chambered swim bladders reached comparable mortality at even lower thresholds, between RPC 1.75 and 2.2^35^. Beirão et al.^65^ showed that depth-acclimated *Pimelodus pictus*, a neotropical silurid species, exhibited significant ruptures already at RPC 1.2–1.5 when decompression was rapid (< 0.1 s). Jones^77^ documented swim bladder ruptures occurring in physoclistous E. perch when the pressure was slowly reduced to 40% (RPC 2.3) of the pressure fish were adapted to. Čada et al.^78^ summarized the available data on different species, and found no mortalities at RPCs below 1.7, and Becker et al.^79^ recommend nadirs at or above 60% of the acclimatization pressure of fish (RPC 1.7) to be safe for a physoclist species, bluegill *Lepomis macrochirus* and different salmonid species. Boys et al.^58^ recommend minimum nadirs of 70% of the acclimatization pressure (RPC 1.44) to effectively protect different physoclistous percids.

These studies, collectively, highlight that even small relative pressure changes can be lethal for species with restricted gas-release capacities such as physoclistous Perciformes and physostomous Cypriniformes, Characiformes and Siluriformes with complex swim bladder morphology. Pflugrath et al.^57^ therefore recommend a conservative approach when aiming to protect a diverse range of species and life stages migrating downstream through turbines in large tropical and neotropical river systems “whereby protection of the most susceptible or vulnerable species ensures protection of all species”.

Depth acclimation strongly influenced barotrauma sensitivity by increasing the RPC experienced during decompression. In our experiments, depth-acclimated E. grayling showed markedly elevated injury rates even at relatively high nadirs when the RPC increased due to prior acclimation to 15 m depth. Rapid decompression to 42 kPa at a rate of pressure change (ROC) of 1,990 kPa s⁻¹ resulted in 94% mortality (RPC 6), but even slow decompression to atmospheric pressure at 3.3 kPa s⁻¹ (RPC 2.5) caused nearly half of the individuals of E. grayling to experience mortal injury. Similarly, 0 + nase acclimated to 15 m depth still experienced 9% mortal injuries even at slow decompression. These findings imply that the physiological capacity of physostomous species to release gas via the pneumatic duct can be overwhelmed once internal gas volume increases under depth acclimation. Earlier observations by Tsvetkov et al.^61^ also described a rate of pressure change of 303 kPa s^− 1^ as lethal for depth-acclimated juvenile grayling; however only two individuals were studied, and no exact values of the RPCs are provided.

Rate of pressure change (ROC) is frequently discussed alongside RPC^42,57^. Natural ROCs that fish typically experience range between 0.17 and 1.69 kPa s⁻¹^61^ whereas Kaplan turbines may generate median ROCs between 750 and 2,500 kPa s⁻¹, with peaks exceeding 4,000 kPa s⁻¹^33^. Brown et al.^20^ reported ROCs between 758 and 3,875 kPa s⁻¹ in large U.S. river hydropower systems, and overall ranges from 62.9 kPa s^− 1^ up to nearly 5,800 kPa s⁻¹^34^. Such rapid pressure changes leave little time for compensatory gas release and are particularly problematic for early developmental stages of fish with already developed parts of their swim bladder and generally for cyprinids with two-chambered swim bladders and percids with limited gas regulation capabilities. Experiments by Tsvetkov et al.^61^ showed that depth-acclimated juvenile roach suffered 100% mortality at ROCs of ~ 304 kPa s⁻¹. Even intermediate ROCs of 10–50 kPa s⁻¹ produced 40–72% mortality in cyprinids, whereas values below 10 kPa s⁻¹ resulted in only 10% mortality. For crucian carp, Meng et al.^62^ found significant injuries at an ROC 2,300 kPa s⁻¹, while Kerr et al.^35^ documented injuries in characids at ROCs between 59 and 341 kPa s⁻¹. Despite these patterns, ROC appears to modulate injury probability around RPC-dependent thresholds rather than serving as an independent driver; this echoes conclusions by Brown et al.^34^ that injuries are ultimately tied more closely to RPC.

Because acclimation pressure depends directly on the depth at which fish have been staying and migrating for a certain time before turbine passage, an improved understanding of their vertical natural distribution is essential for predicting barotrauma risk. Juvenile individuals e.g. during downstream migration in autumn/winter^80^ or adult individuals e.g. after spawning^81–83^ might prefer to migrate downstream at greater depths, while most larval fish drift near the surface, typically within the upper 0–1 m^84^. However, cyprinid larvae have been found down to ~ 4 m^85^ and grayling L1/L2 larvae (without a sim bladder at this stage) at 2–3 m^86^. Even such modest differences can substantially alter barotrauma risk in fish with developed swim bladders^34,87^.

While migration distances and proportions of adult fish passing through turbines fish might be partly known^88^, migration patterns and drift distances of juveniles and larvae remain poorly documented for most species. E.g. for common nase relatively short distances were documented when suitable rearing habitats exist near spawning grounds^89^, whereas grayling larvae may drift up to 30 km^86^. For rivers with multiple hydropower stations, cumulative turbine exposure is therefore likely for at least a fraction of migrants from different life stages. Given current knowledge gaps, conservative assumptions and species- or guild-specific monitoring are essential for reliable risk assessment.

Operational analyses showed that turbine management strategies can meaningfully reduce barotrauma risk. Full-load operation produced substantially higher injury probabilities than partial-load operation; for cyprinids, mortal injury probabilities decreased from around 8% during full load to below 2% during partial load. Strategic timing of partial-load operation during peak larval and juvenile migration could therefore reduce barotrauma-related mortality, although blade-strike risk for larger fish may increase under partial-load conditions^90^. Long-term solutions likely require a combination of operational adjustments, structural modifications (e.g. fish-friendly turbine designs, protection and bypass structures), and pressure-profile optimization to reduce extreme pressure conditions. Integrating barotrauma considerations into the design and environmental assessment of new RoR-HPPs will be important to mitigate impacts on fish populations.

Overall, this study showed that swim bladder morphology, developmental stage, depth acclimation, and turbine-induced pressure dynamics interact to determine barotrauma risk. The particularly high vulnerability of two-chambered physostomous fish such as many Cypriniformes and Characiformes implies that large portions of global freshwater fish diversity may be at similar risk, an issue that has thus far been underestimated.

Future investigations should therefore incorporate realistic hydraulic turbine models, broader RPC–ROC combinations, and a wider diversity of species and ontogenetic stages dominantly migrating downstream through turbines, including adults and depth-acclimated individuals. Long-term and delayed mortality, cumulative impacts from repeated turbine passage, interactions with predation risk, and distributions of fish within the water column during migration all require further study. Such research is necessary to ensure reliable impact assessments and inform evidence-based mitigation strategies that support sustainable hydropower development while conserving and restoring fish populations worldwide.

## Methods

### Species studied

The species under study were selected to represent fish communities inhabiting medium sized rivers in Europe based on their differing swim bladder morphologies and known downstream migrations during different developmental stages^80,91–94^.

Four species were studied, common nase *Chondrostoma nasus*^92,95^ and roach *Rutilus rutilus*^85,92,96,97^ as physostomous cypriniform species with a two-chambered swim bladder^53^, European grayling *Thymallus Thymallus*^86^ as physostomous salmonid species with a single-chambered swim bladder^73,74,98^ and European perch *Perca fluviatilis*^60,99,100^ as physoclistous percid species with a single chambered swim bladder^73,101^.

The common nase *C. nasus* belongs to the family Cyprinidae, subfamily Leuciscinae, one of the most diverse monophyletic families within the Holarctic Cypriniformes. This family has a broad geographic distribution and notable endemism, with common nase having by far the largest distribution range among the genus *Chondrostoma*^102,103^. Due to its sensitivity to environmental changes across various spatial and temporal scales, particularly related to habitat use and migratory patterns, the nase, a rheophilic cyprinid, has become a flagship species for monitoring and conservation efforts in large European river systems^104^. Its conservation status is a matter of concern^105,106^.

Roach *R. rutilus* is a typical ubiquitous widespread cyprinid species with a two-chambered swim bladder in Europe and Asia with high invasive potential^103^. It’s drift behavior at larval and juvenile stages has been extensively studied^107^, and it has been used as an indicator species for modelling recent stock declines in rivers^108^.

European grayling *T. thymallus* is a member of the salmonid family, subfamily Thymallinae with about 15 species and circumpolar distribution, most diverse in North East Asia^103,109^, with increased conservation concerns in Europe^105,109^.

European perch *P. fluviatilis* is one of the most common freshwater fishes in the world with a native range across Europe and Asia^110^ and closely related and strongly resembling the yellow perch *Perca flavescens* of North America. E. perch is highly relevant for recreational fisheries and aquaculture and is therefore considered as an important representative of physoclistous species.

Although both, European grayling and common nase, are considered as endangered in specific areas^105^, the IUCN only lists nase as near threatened^111^, while roach, E. grayling and E. perch are considered as least concern^112–114^.

Developmental stages studied were defined according to Penáz^73^ and were L1 ( TL = 12.0 ± 0.4 mm) and 0+ (TL = 41.9 ± 7.8 mm) for nase, L6/J1 (TL = 24.3 ± 1.5 mm) and 0+ (TL = 83.6 ± 7.5 mm) for roach, L1/L2 (TL = 18.3 ± 0.6 mm) and 0+ (TL = 97.5 ± 9.0 mm ) for E. grayling and L1/L2 (TL = 6.3 ± 0.2 mm), L6/J1 (TL = 21.4 ± 1.6 mm) and 0+ (TL = 39.8 ± 4.5 mm ) for E. perch. L refers to larval stages, J to early juvenile stages, and 0 + to young-of-the-year fish in the autumn of their first year of life, with some fish being at the transition between stages^73^.

### General experimental setup

The experiments were conducted in the experimental hall at the Graz University of Technology, Austria between 20.5.2020 and 24.6.2021. The setup included a large basin (2.5 m diameter, 0.9 m depth, 4.4 m³ volume) continuously fed with fresh drinking water being circulated and oxygenated by aerators supplying water to the whole system. For keeping the fish, the system comprised three large tanks (1.5 m diameter, 0.9 m depth, 1.6 m³ volume each), two rectangular swimming flumes, and six round tanks (60 cm diameter, 40 cm height, 80 l each). Two smaller rectangular tanks cooled by a surrounding water circuit were used for fish recovery after the experiments conducted under anesthetization containing 5 mg l^− 1^ Tricaine (MS 222). Water quality parameters (temperature, pH, conductivity, oxygen saturation, nitrate, nitrite, ammonium) were monitored every 4 h to ensure fish welfare over the whole duration of the experiments. Water temperatures ranged between 7.4 and 22.9 °C depending on the season of the year, O_2_ saturation of the water circulating in the system ranged between 90 and 101%.

### Barotrauma chamber

The main element of the experimental setup was a newly designed barotrauma chamber that was connected to the water circulation system to run the systematic depressurization trials^72^. The chamber consists of a T-shaped pipe section with a diameter of 300 mm with a volume of approximately 75 l and has a 50 mm thick Plexiglas panel at the front end and is sealed on the other end with a lid. This permanently bolted lid contains pressure and temperature sensors as well as the linear actuator. The linear actuator has a usable stroke of 96 mm and features an internal brake that holds the piston in the selected position. The chamber itself is connected to a water supply tank located beneath it with a volume of approximately 200 l. Water can be circulated between the chamber and the supply tank at a delivery rate of 7–20 l min^− 1^. The purpose of the buffer tank is to supply fresh water to the barotrauma chamber during acclimation trials, to increase the pressure and to introduce anesthetics. The buffer tank is also equipped with an optional heating system with temperature control and an air supply system to maintain constant oxygen levels. The pressure curve applied to fish in the barotrauma chamber is determined by a moveable piston with linear actuator. The maximum achievable pressure during acclimation is 400 kPa (about 30 m water depth). The chamber can therefore reproduce the pressure patterns found in Kaplan turbines of all Austrian Run-of-River Hydropower Plants (RoR-HPPs) with median rates of pressure change (ROCs) between 750 and 2,500 kPa s⁻¹ and peaks above 4,000 kPa s⁻¹, and minimum nadirs as low as 5 kPa (mean minimum nadir: 24 kPa)^33^. The chamber can match these conditions by producing pressure drops from 400 kPa down to 15 kPa in less than 0.1 s, at rates exceeding 3,850 kPa s⁻¹^72^ (see **Supplementary Fig. 1**). A representation of the main components of the chamber is shown in **Supplementary Fig. 2**, and a detailed description its performance can be found in Schneider et al.^72^.

The pressure increase in the chamber can be achieved either via the circulating pump or directly via the piston extending into the chamber. Before a pressure drop occurs, the pump is hermetically separated from the chamber by a seal. The desired pressure drop (variable acceleration, velocity, and extended length) can then be achieved by the software-controlled piston retracting it from the chamber. The pressures in the chamber were measured using a piezoresistive pressure transmitter (Keller PR21Y), with a pressure range between 0 and 500 kPa. A custom-developed software controls the servo motor (EMMS-AS-100-M-HS-RMB), which, via an electrically driven spindle, converts the rotary motion into a linear motion of the piston rod. The maximum piston speed is 960 mm s^− 1^, the acceleration is 6 m s^-2^ , and the deceleration is 25 m s^-2^.

Since the pressure gradient in the chamber is highly dependent on the amount of air present, repeated tests with varying air volumes and nadirs were conducted during the chamber’s development, as well as prior to experiments with fish, to ensure the target nadirs could be achieved. A crucial factor for the successful execution of pressure experiments with fish was therefore the proper handling of the chamber to prevent unintentional residual air. To minimize air in the chamber all air bubbles were carefully removed before each experiment. Because fish of different species and sizes introduce air through their swim bladders, the volume of this air was estimated beforehand to adjust the chamber controls accordingly. Consequently, preliminary tests with defined air volumes were always performed immediately before fish experiments to ensure the controls were properly configured.

### Fish handling, acclimation, selection criteria and blinding

Fish larvae and juveniles were delivered from a hatchery in plastic bags containing water and air one or two days before the experiments. Fish were then acclimated in well-aerated water in the rectangular or round holding tanks. Fish were fed the same diet they were accustomed to from the hatchery. To minimize mechanical stress and potential pre-experiment injuries, fish larvae were first transferred from the holding tanks into Plexiglas cylinders using a measuring cup, avoiding the use of nets, and then carried to the experimental chamber. The Plexiglas cylinders (12.5 width and 20 cm length) were covered on one side with a fixed 250 μm mesh and could be closed by a removable lid on the other side. Lids used had either also a 250 μm mesh (for the experiments) or were fully closed (for the transfer of fish).

The main inclusion criteria were that the fish were in the developmental state required for the experiments and did not show any sign of injury and abnormal behavior. The person who selected the fish for the trials did not know about the pressure gradient applied by two technicians who were running the experiments with the barotrauma chamber and noted all the details of the experiment (e.g. real nadir achieved) in a separate file.

### Determination of anesthesia concentration

For the anesthesia of the fish, MS 222 (Tricaine Methane Sulphonate 100%) from Pharmaq AS (Norway) was used, which was buffered in a ratio of 1:2 with 99.95% pure sodium bicarbonate (WoldoHealth, Germany)^115–117^. The required amounts were weighed using a precision scale from Kern (ABT 220-5 DM, KERN & SOHN GmbH, Germany). Five larvae per experiment were anesthetized and observed in a Petri dish under a binocular microscope, while juvenile fish were anesthetized in three liters of anesthetic solution in glass aquaria (30 × 20 × 20 cm, 12 l). The number of anesthetized larvae/juvenile fish and their condition and behavior after three minutes of anesthesia, eight minutes under anesthesia, and ten minutes of recovery time were noted, and the number of dead larvae was recorded immediately after the experiment, after one hour, and after 24 h. All experiments were replicated twice.

The goal was to determine the concentration of Tricaine (MS 222) in mg l^− 1^ required to achieve deep anesthesia (Phase III Stage 2^118,119^ within three minutes, maintain it for eight minutes, and allow recovery within ten minutes. The anesthetic concentration was adjusted based on the sensitivity of the fish. The final concentrations ranged from 5 mg l^− 1^ to 90 mg l^− 1^ depending on species and developmental stage (**Supplementary Table 3**).

### Potential effects of anesthesia

For the experimental determination of a potential effect of anesthesia on the results^65^, fish were divided into groups of 70 individuals per treatment (anesthetized vs. non-anesthetized). Fish were placed in the Plexiglas cylinders and then introduced into the barotrauma chamber filled with water containing the appropriate concentration of anesthetic. Control groups were treated similarly but without pressure changes. All individuals of each group were simultaneously subjected to the treatment. These experiments were run for E. perch (L1/L2, L4, 0+), E. grayling (L1/L2, 0+) and roach (0+) with target nadirs of 15, 50 and 70 kPa. To statistically evaluate differences with the possibility of detecting differences between 0.9:0.7 = 0.1:0.3 in mortality, the group size was set at *n* = 70, using equal sample sizes and Fisher’s exact test with a power of 80%. Since the real nadir values achieved during the experiments slightly differed between comparison groups, additionally a binary logistic regression to assess potential effect of anesthesia on the likelihood of mortal injuries was performed, using the anaesthetization treatment as categorical variable and the nadir pressure as continuous variable.

### Atmospheric pressure trials

Experiments with fish acclimated to atmospheric pressure to create the dose-response curves were conducted for nase (L1, 0+), roach (L6/J1, 0+), E. grayling (L1/2, 0+) and E. perch (L1/L2, L6/J1, 0+) targeting nadirs of 15, 30, 40 and 60 kPa. Ten fish per trial were transferred to Plexiglas cylinders, put into the barotrauma chamber containing the pre-determined concentration of MS 222, and subjected to pressure drops. Starting at atmospheric pressure of about 101 kPa, the pressure curves simulated represented a turbine passage of a fish through a medium-sized low-head RoR-HPP equipped with a Kaplan turbine and a maximum water depth of about 15 m. The pressure was first increased to 251 kPa over 20–25 s, followed by a sharp pressure drop to the selected target nadir within 0.1–0.2 s. The resulting ROCs ranged from 1,180 to 1,910 kPa s^− 1^ with slower rates occurring at lower nadirs. Nadir durations ranged from 0.1 to 0.2 s, although 40% of nadirs targeting 15 kPa lasted between 0.2 and 1.5 s, with only five exceeding 0.4 s. After each nadir, the pressure was increased to approximately 180 kPa, followed by a transition back to atmospheric pressure over 12–16 s. For details on the general performance of the barotrauma chamber see Schneider et al.^72^. Pressure values were estimated by assuming a mean atmospheric pressure of 101 kPa and adding the hydrostatic pressure of a 15 m water column (~ 150 kPa). A water density of 1,000 kg m⁻³ was used, with standard gravity and without adjustments for altitude.

Typically, three replicates were conducted, in a few cases only two replicates (roach L6/J1 at target nadir 40 kPa) or due to the lack of fish, no experiments could be run (roach L6/J1 at target nadir 60 kPa). Some experiments were repeated, as it was not clear if the technical parameters were met but were later used in the analysis when the data proved valid. That is why for nase L1 at target nadir 15 kPa and L1/L2 E. grayling at target nadir 60 kPa four replicates were available.

### Calculation of dose-response curves

For the calculation of the dose-response curves linking pressure changes to probabilities of mortal injuries, only the systematic trials using fish acclimated to atmospheric pressure were used. The most relevant parameter in predicting barotrauma-related injuries was expected to be the ratio of pressure change (RPC)^22^. The RPC is the ratio between the pressure of acclimation (when fish are neutrally buoyant which is achieved by means of regulating the swim bladder volume) divided by the lowest pressure of exposure (the nadir). Using the natural log of the pressure-change ratio (LRP) allows the combination of the pressure at depth of acclimation and nadir pressure into a single term, with LRP = log(Pressure at Acclimation depth P_A_/Pressure at Nadir P_N_). A logistic regression can be then used, to calculate the model parameters b_0_ (intercept) and b₁ for the dose-response curve between the LRP or LN(P_A_/P_N_) and the probabilities of mortal injuries^34^. For predicting mortal injuries at given LRPs the Eq. 1 can be used^22,34^:

$$\:P\left(X\right)=\:\frac{{e}^{b0+b1*\left(LN\left(\frac{PA}{PN}\right)\right)}}{1+{e}^{b0+b1*\left(LN\left(\frac{PA}{PN}\right)\right)}}$$

**Eq. 1**: Calculation of the probability of fish injury based on the log-transformed ratio of acclimation pressure to nadir pressure; it can be also expressed as *P(X)* = exp(b0 + b1*LN(P_A_/P_N_))/(1 + exp(b0 + b1*LN(P_A_/P_N_))).

To consider handling mortality for the creation of the dose-response curves, the mean of the dead fish found in the control groups were subtracted from the mortally injured fish in the respective experiments, or the number of dead fish was set to zero.

### Acclimation trials

To test the effects of acclimatization on barotrauma-related injuries, 70 individuals of nase, roach, E. grayling and E. perch were directly put into the barotrauma chamber and then acclimated for 18 to 24 h until they achieved buoyancy. During the acclimation process, pressure was gradually increased from atmospheric pressure (101 kPa) to the target value of 251 kPa over a period of one hour, while maintaining a flow rate of 8–20 l min^− 1^. Once the target pressure of 251 kPa was reached, it was held constant in the chamber, and a continuous flow of water, enriched with atmospheric oxygen from the supply tank, was maintained. For the physostomous species an air bubble was kept in the chamber to allow for gulping air to fill the swim bladder. Shortly before the experiment, the chamber was completely deaerated by carefully opening the valve located on the riser pipe. Once the chamber was free of air, the anesthetic was added to the supply tank and mixed with the aerated water in the chamber over the course of approximately 7 min using the circulation pump at a flow rate of 20 l min^− 1^. After mixing, the flow between the supply tank and the chamber was closed. The fish were then exposed to either a 45-second pressure drop to atmospheric pressure (ROC: 3.3 kPa s^− 1^, control group) or a rapid pressure drop of 0.1–0.2 s to specific nadirs: 15 kPa (perch 0+), 29 kPa (nase 0+), 44 kPa (roach 0+), 42–44 kPa (E. grayling 0+). The pressure drops had ROCs ranging between 1,180 kPa s^− 1^ (perch 0+) and 2,070 kPa s^− 1^ (roach and E. grayling 0+), with slower rates occurring at lower nadirs (**see Supplementary Table 4**). Nadir durations were 0.5–1 s, followed by a rise to higher pressure and then a return to atmospheric pressure over approximately 12 s.

### Fish treatment experiments, euthanasia and necropsy

After the pressure gradients were applied, the barotrauma chamber was opened, and the Plexiglas cylinders containing the anesthetized fish were carefully removed. Each cylinder was then covered on one side with a closed lid to retain sufficient water for the transfer to a pool containing 5 mg l^− 1^ MS 222 for pain-free recovery over a duration of 10–12 min. Fish exhibiting abnormal behavior or physical deformities were euthanized with 0.5 g l^− 1^ MS 222 to reduce pain and distress and subsequently examined under a microscope for injuries. Additionally, fish behavior and mortality were observed during a subsequent 24-hour post-experiment period, where fish were held in numbered cages together with a note on the day and time of the experiment. Dead fish or fish exhibiting abnormal behavior or physical deformities during this time were extracted and euthanized with 0.5 g l^− 1^ MS 222 and subjected to the injury examination procedure. Finally, after 24 h, all remaining experimental animals including control fish were euthanized and subjected to necropsy. This ensured a painless endpoint throughout the entire experiment. The person who observed the fish over the duration of 24 h did not know anything about the pressure gradient applied during this respective experiment. To document the injuries caused by pressure changes, score sheets were created listing the known and expected types of damage, such as internal bleeding, ruptured swim bladders, exophthalmia (protruding swim bladder and eyes), blood or bile exiting from the throat, and gas bubbles (embolisms) in the gills and fins^20,34,76^. Additional types of injuries were identified during the study, such as ruptured abdominal walls or rupture of the peritoneum in nase. All experimental fish were systematically examined for these signs of injuries under a binocular microscope (Olympus SZ 40) or with a camera connected to a computer (Canon Eos 6d, Canon EF 50 mm f/2.5 Compact Macro or Canon MP-E 65 mm f/2.8 1-5x Macro, mounted on Giottos MTL 8361B, with Wallimex pro Niova 900 Plus Daylight as a light source). The lengths of the larvae were measured using a binocular microscope (S63T Trinocular Pod (8-50x), China) with an attached digital camera (ProgRes^®^ CT3, Jenooptik, Germany), while the lengths of the juvenile fish were measured with a ruler. Larval and early juvenile fish could be inspected for external and internal injuries without incision from outside due to their transparency, in 0 + individuals the body cavity was additionally incised and opened in a white laboratory tray containing water to check for escaping gas bubbles, followed by an internal necropsy without water coverage. The fish physiology expert who was doing the necropsy did not know about the pressure gradient applied when receiving the fish for necropsy.

### Determination of mortally injured fish

To determine the number of mortally injured fish, in an initial step dead fish and fish with aberrant behavior (both immediately and during follow-up observations up to 24 h) were categorized as “functionally dead” and examined for injuries. To account for possible delayed mortalities beyond the 24-hour observation period due to clearly identifiable injuries in fish that were still alive and showed no signs of abnormal behavior up to that point, a so-called “mortal injury metric”^76^ was calculated, following an approach applied in similar studies^34,57^. The aim was to identify injuries that were significantly associated with a high probability of death or abnormal behavior. All fish subjected to pressure changes during the atmospheric pressure trials, pre-experiments and acclimation trials with fast and slow pressure changes were used in the calculations, control fish were not included.

**Fig. 3:**
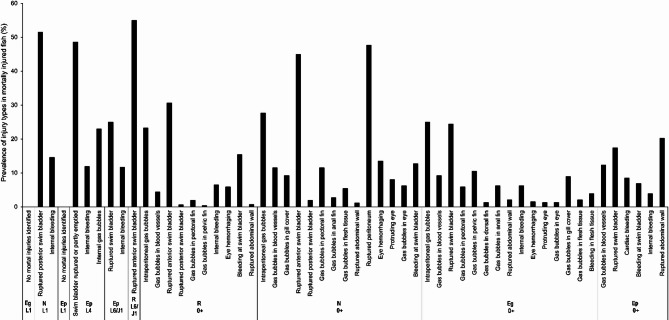
Prevalence of injury types in mortally injured fish per species and life stage (Eg = European grayling, N = nase, Ep = European perch, R = Roach; L1 = larval stage 1, L4 = larval stage 4, L6/J1 = larval stage 6/transition to juvenile stage 1, 0 + = young-of-the-year fish in the autumn of their first year of life) as identified by Fisher’s exact test and Odd’s ratio as a first step for the calculation of the “mortal injury index”^76^ based on the total number of experimental fish subjected to pressure changes (no control fish were included, see Table 4 for more details, and also **Supplementary Table 3** for the different experiments conducted). In L1/2 E. perch and E. grayling no injuries could be identified, only dead and abnormal fish were counted. Note that the percentages of documented injuries in mortally injured fish cannot be directly compared between the different life stages of species, as not for all life stages and species the same experiments were conducted; however for each species and life stage the percentages of the documented injuries enable to determine their susceptibility to specific injury types.

The calculation was performed in two steps. First, Fisher’s exact test and the Odd’s ratios were calculated using contingency tables to identify injuries that were significantly associated with death or abnormal behavior in larvae and juvenile fish at a significance level of *P* < 0.05 (Fisher’s exact test) and an odds ratio > 1 (Fig. 3; Table 4). The odds ratio generally indicates how much more likely it is that fish with a specific injury are dead or show abnormal behavior.

Subsequently, those injuries that were clearly associated with death or abnormal behavior were subjected to a stepwise logistic regression to determine the significance of each injury for predicting death or abnormal behavior. Fish that were still alive and externally normal after 24 h but exhibited these injuries were subsequently classified as “assigned mortally injured after dissection” and were grouped together with dead and behaviorally abnormal fish (category “functionally dead”) under the collective classification “mortally injured”. It has to be mentioned that in two cases (ruptured abdominal wall in 0 + nase, *P* = 0.051 Odd’s ratio = 8.86, and intraperitoneal gas bubbles in 0 + roach, *P* = 0.055 and Odd’s ratio = 1.53), the calculated p-values were interpreted as clear trends and were included in the logistic regression analysis. However, only the parameter “intraperitoneal gas bubbles” in roach was evaluated as significant, while the abdominal wall rupture in nase was not further used.

**Fig. 4:**
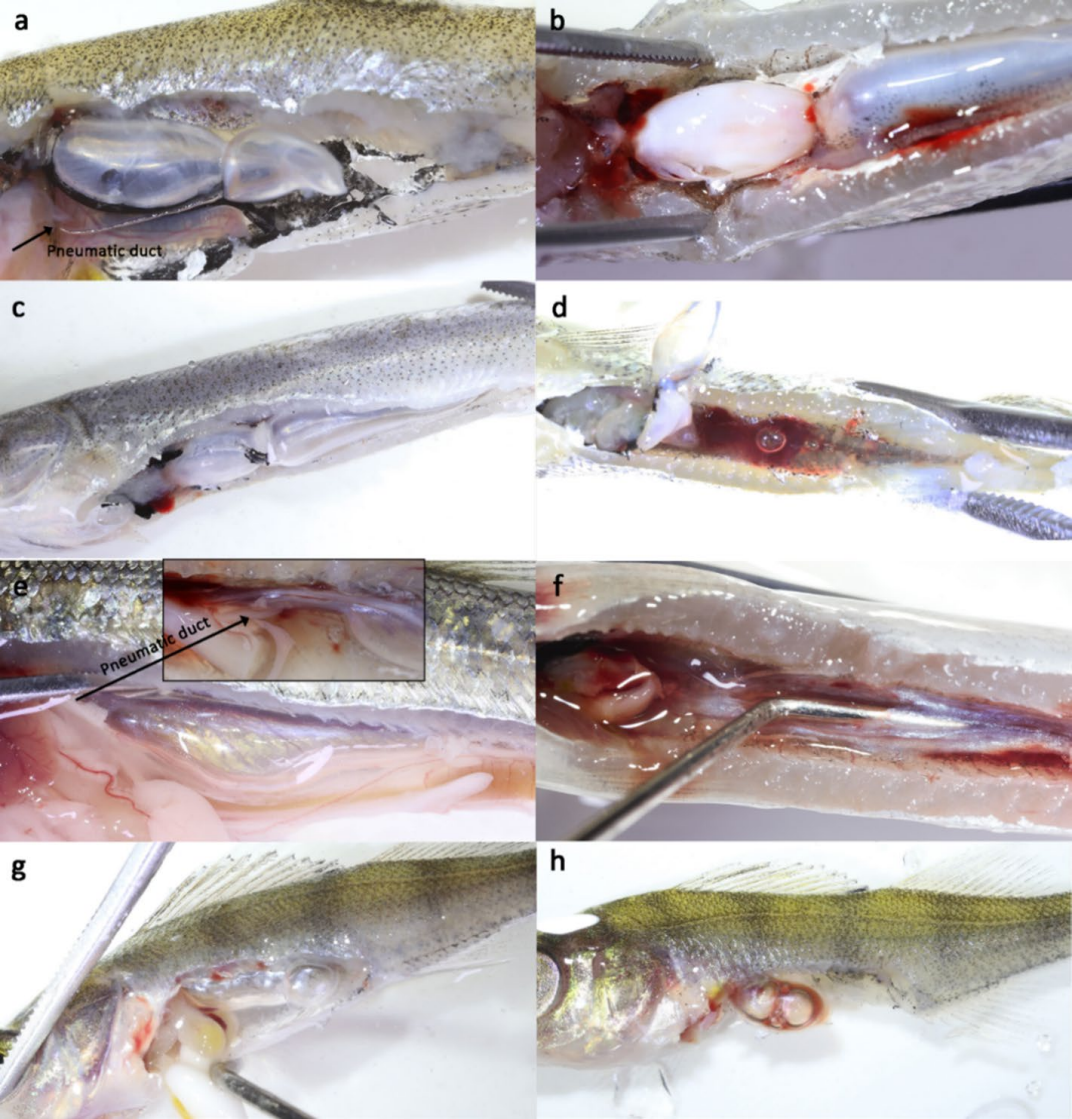
Intact (left) and ruptured anterior swim bladders (right) in 0 + of physostomous roach (a, b) and common nase (c, d) with intraperitoneal gas bubbles, intact and ruptured swim bladder in 0 + of physostomous E. grayling (c, d) and intact and ruptured swim bladder in 0 + of physoclistous E. perch (g, h) with internal bleeding; arrows are indicating the pneumatic duct in roach (a) and E. grayling (e).

**Fig. 5:**
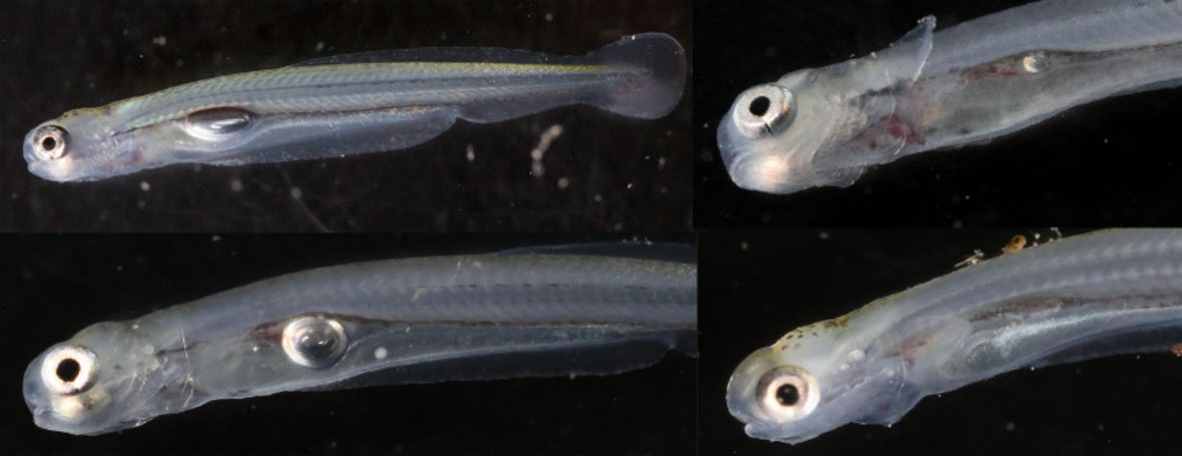
Intact posterior swim bladder (upper left) and ruptured posterior swim bladders with associated internal bleeding in L1 nase with different grades of remaining gas in the body cavity after barotrauma; similar degrees of barotrauma-related injuries are displayed in Tsvetkov et al.^61^. Note that nase are filling the posterior part of their swim bladder first after hatching^53^, which is considered the main reason for their downstream dispersal^95^.

Ruptured swim bladders, even when not included in the logistic regression predicting death or aberrant behavior within 24 h although identified as significantly associated with death or abnormal behavior by Fisher’s exact test, were considered as mortal injuries in 0 + E. grayling, L6/J1 E. perch, 0 + E. perch (see Fig. 4 for a comparison of intact and ruptured swim bladders in the 0 + individuals of the studied species) and L1 nase (Fig. 5). This was justified by literature as ruptured swim bladders typically are also causing the presence of conspicuous free gas bubbles in the abdominal cavity indicating that these fish might be subjected to delayed mortality (> 24 h), or that behavioral abnormalities or inflammations may later occur in the wild, exposing the fish to increased predation^20,75^. For example, Mueller et al.^75^ showed a high rate of salmonids with free intraperitoneal gas experiencing delayed mortality after turbine passage. Although Bellgraph et al.^120^ have shown that surgically incised swim bladders in salmonids can heal, in the case of the documented irregularly ruptured or torn swim bladders, this is considered unlikely.

Summarizing, mortal injuries identified by the described procedure (Fisher’s exact test, subsequent logistic regression, consideration of ruptured swim bladders as mortal injuries) were (Table 4):

- L1 nase: ruptured (posterior) swim bladder, internal bleeding.
- L1 E. perch: no mortal injuries could be identified.
- L4 E. perch: swim bladder ruptured or partly emptied.
- L6/J1 E. perch: ruptured swim bladder, internal bleeding.
- L6/J1 roach: ruptured anterior swim bladder.
- 0 + roach: ruptured anterior swim bladder, intraperitoneal gas bubbles, gas bubbles in blood vessels, eye hemorrhaging, ruptured abdominal wall.
- 0 + nase: ruptured anterior and/or posterior swim bladder, gas bubbles in blood vessels and gill cover, rupture of the peritoneum, eye hemorrhaging, protruding eye.
- 0 + E. grayling: ruptured swim bladder, intraperitoneal gas bubbles, gas bubbles in blood vessels, pectoral and pelvic fins, ruptured abdominal wall.
- 0 + E. perch: ruptured swim bladder, gas bubbles in blood vessels, ruptured abdominal wall, internal bleeding, cardiac bleeding.

**Table 4:** Summary of the documented significant injuries with total number of experimental fish (see also **Supplementary Table 3** for the different experiments conducted) subjected to pressure changes (no control fish were included), divided into dead/abnormal fish and fish determined as mortally injured when additionally considering fish living and being normal after 24 h, but showing the significant injuries determined by the mortal injury index calculation. As in L1/L2 individuals of E. perch and E. grayling no injuries could be visually identified, only the dead and abnormal fish were counted. Note that the percentages of mortally injured individuals cannot be compared between the different life stages of species, as not for all life stages and species the same experiments were conducted. For each species the percentages of documented injuries enable to determine the susceptibility of the life stage to the documented injury type.

Species / Life stage	Total *n* number of individuals subjected to pressure changes	Total % fish dead or abnormal in 24 h	Significant injuries*	% fish dead or abnormal in 24 h with this injury	Fisher’s exact test sign.	Odd’s ratio	Injuries in LogReg**	Stepwise LogReg. Sign. (*n*.s. = not significant)	Total % mortally injured fish with this injury	Total % mortally injured fish
E. grayling L1	410	0.7	No mortal injuries identified							0.7
Nase L1	130	7.7	Ruptured swim bladder	6.9	0.011	1.862	-	n.s.	51.5	53.8
			Internal bleeding	5.4	< 0.001	7	Internal bleeding	< 0.001	14.6	
E. perch L1	260	33.5	No mortal injuries identified							33.5
E. perch L4	278	44.6	Swim bladder ruptured or partly emptied	39.9	< 0.001	5.744	Swim bladder ruptured or partly emptied	< 0.001	48.6	55.8
			Internal bleeding	8.6	0.017	1.987	-	n.s.	11.9	
			Internal gas bubbles	18.3	< 0.001	4.872	-	n.s.	23	
E. perch L6/J1	120	12.5	Ruptured swim bladder	10	< 0.001	4.667	-	n.s.	25	27.5
			Internal bleeding	10	< 0.001	42	Internal bleeding	< 0.001	11.7	
Roach L6/J1	80	38.8	Ruptured anterior swim bladder	37.5	< 0.001	3.387	Ruptured anterior swim bladder	< 0.001	55	60
Roach 0+	540	8.7	Intraperitoneal gas bubbles	3	0.055	1.526	Intraperitoneal gas bubbles	< 0.001	23.3	33.3
			Gas bubbles in blood vessels	3.5	< 0.001	39.86	Gas bubbles in blood vessels	0.006	4.4	
			Ruptured anterior swim bladder	8.1	< 0,001	3.814	Ruptured anterior swim bladder	< 0.001	30.6	
			Ruptured posterior swim bladder	0.6	0.002	31.532	-	n.s.	0.6	
			Gas bubbles in pectoral fin	1.9	< 0,001	105.106	-	n.s.	1.9	
			Gas bubbles in pelvic fin	0.4	0.021	21.021	-	n.s.	0.4	
			Internal bleeding	1.5	0.012	2.797	-	n.s.	6.5	
			Eye hemorrhaging	4.4	< 0,001	31.468	Eye hemorrhaging	< 0.001	5.9	
			Bleeding at swim bladder	4.4	< 0,001	3.449	-	n.s.	15.4	
			Ruptured abdominal wall	0.6	0.002	31.468	Ruptured abdominal wall	0.005	0.7	
Nase 0+	260	25.4	Intraperitoneal gas bubbles	11.5	< 0.001	2.1	-	n.s.	27.7	55
			Gas bubbles in blood vessels	11.5	< 0.001	88.636	Gas bubbles in blood vessels	< 0.001	11.5	
			Gas bubbles in gill cover	9.2	< 0.001	70.909	Gas bubbles in gill cover	0.012	9.2	
			Ruptured anterior swim bladder	22.3	< 0.001	2.89	Ruptured anterior swim bladder	0.002	45	
			Ruptured posterior swim bladder	1.5	0.016	11.758	Ruptured posterior swim bladder	0.021	1.9	
			Gas bubbles in pectoral fin	10	< 0.001	19.106	-	n.s.	11.5	
			Gas bubbles in anal fin	2.7	< 0.001	20.682	-	n.s.	2.7	
			Gas bubbles in flesh tissue	3.8	< 0.001	7.348	-	n.s.	5.4	
			Ruptured abdominal wall	1.2	0.051	8.864	-	n.s.	1.2	
			Ruptured peritoneum	20	< 0.001	2.123	Rupture of the peritoneum	0.008	47.7	
			Eye hemorrhaging	11.5	< 0.001	17.636	Eye hemorrhaging	< 0.001	13.5	
			Protruding eye	7.3	< 0.001	27.924	Protruding eye	0.009	8	
			Gas bubbles in eye	6.2	< 0.001	47.273	-	n.s.	6.2	
			Bleeding at swim bladder	5.4	0.017	2.166	-	n.s.	12.7	
E. grayling 0+	610	18.4	Intraperitoneal gas bubbles	16.9	< 0.001	9.16	Intraperitoneal gas bubbles	< 0.001	25	29
			Gas bubbles in blood vessels	9.2	< 0.001	249.5	Gas bubbles in blood vessels	< 0.001	9.2	
			Ruptured swim bladder	15.6	< 0.001	7.822	-	n.s.	24.4	
			Gas bubbles in pectoral fin	5.9	< 0.001	160.393	Gas bubbles in pectoral fin	0.032	5.9	
			Gas bubbles in pelvic fin	10.5	< 0.001	285.143	Gas bubbles in pelvic fin	< 0.001	10.5	
			Gas bubbles in dorsal fin	1.3	< 0.001	35.643	-	n.s.	1.3	
			Gas bubbles in anal fin	6.2	< 0.001	169.304	-	n.s.	6.2	
			Ruptured abdominal wall	2.0	< 0.001	53.357	Ruptured abdominal wall	0.057	2.1	
			Internal bleeding	4.6	< 0.001	12.45	-	n.s.	6.2	
			Eye hemorrhaging	1.3	< 0.001	35.571	-	n.s.	1.5	
			Protruding eye	1.3	< 0.001	35.643	-	n.s.	1.3	
			Gas bubbles in eye	1.3	< 0.001	35.643	-	n.s.	1.3	
			Gas bubbles in gill cover	8.5	< 0.001	115.607	-	n.s.	8.9	
			Gas bubbles in flesh tissue	2.1	< 0.001	57.92	-	n.s.	2.1	
			Bleeding in flesh tissue	2.3	< 0.001	6.225	-	n.s.	3.9	
E. perch 0+	540	28.3	Gas bubbles in blood vessels	10.0	< 0.001	12.417	Gas bubbles in blood vessels	< 0.001	12.3	38.1
			Ruptured swim bladder	11.7	< 0.001	5.14	-	n.s.	17.4	
			Cardiac bleeding	7.0	< 0.001	12.015	Cardiac bleeding	0.009	8.5	
			Bleeding at swim bladder	4.4	< 0.001	4.67	-	n.s.	6.9	
			Internal bleeding	3.7	< 0.001	50.588	Internal bleeding	< 0.001	3.9	
			Ruptured abdominal wall	13.9	< 0.001	5.58	Ruptured abdominal wall	< 0.001	20.2	

### Collection of pressure data for Kaplan turbines

**Fig. 6:**
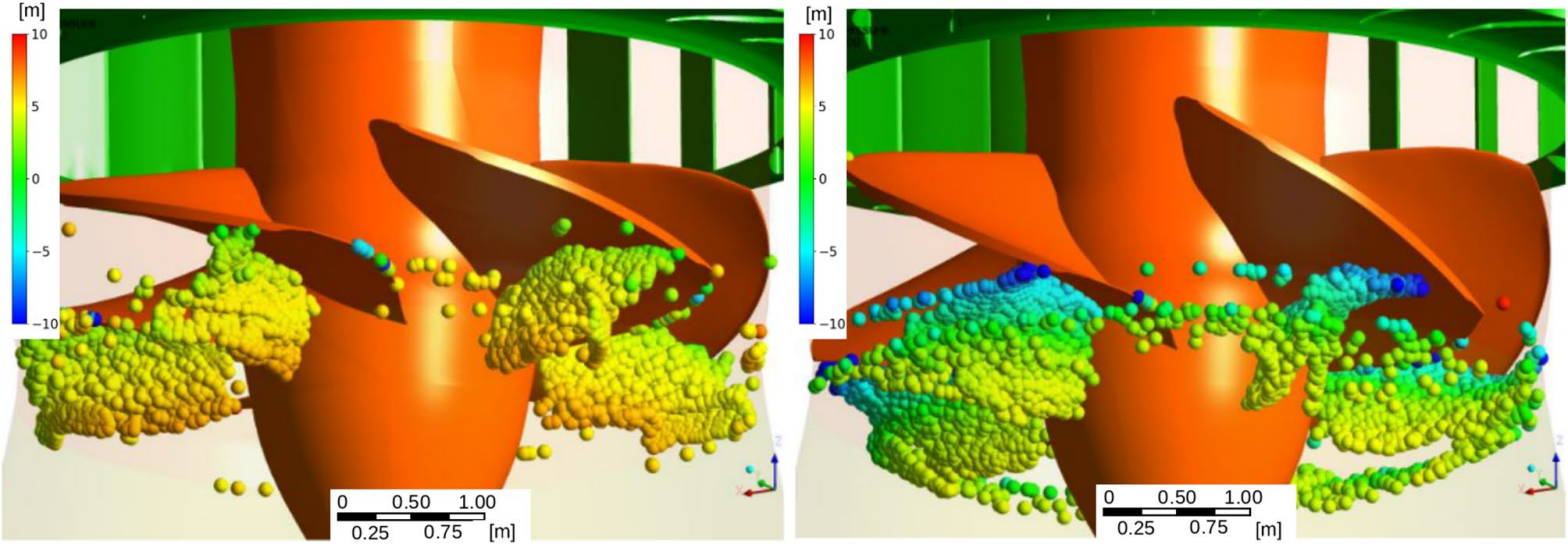
Location of lowest pressures on particle tracks (*n* = 5000): left – partial load operation, right – full load operation (Benigni et al.^122^, modified); summarized results of the simulated nadir distributions can be found in **Supplementary Table 2**).

To determine the pressure curves for programming the barotrauma chamber, a specifically developed Barotrauma Detection System (BDS) were used at Kaplan turbines with discharges between 100 and 275 m³ s^− 1^ typical for medium sized rivers **(Supplementary Table 5)**^33,121^. To determine a statistically relevant distribution of nadirs potentially experienced by fish drifting through the investigated Kaplan turbines, numerical simulations using Computational Fluid Dynamics (CFD) modelling were conducted (Fig. 6)^122^. The solver software applied was the commercial CFD code Ansys CFX V17.1. Hydraulic traces of *n* = 5000 particles were modelled, and the lowest nadirs and their positions along the track were recorded. Pressures were lowest directly in the area below the turbine blades and were lower near the blade tips as compared to the central area (Fig. 4). The lowest simulated pressures along the particle ranged between 0 and 169.1 kPa in full load condition, and between 2.6 and 175.8 kPa in partial flow condition, depending also on the position of particle entering the upper boundary condition (turbine). The statistical distribution of lowest nadirs along particle tracks (a summary of the simulated nadir distributions can be found in **Supplementary Table 2**) were finally used in combination with the logistic biological dose-effect curves to calculate the percentage of mortally injured fish during turbine passage. The logistic equations were applied starting below nadir pressures of 60 kPa (approximately 0.5 LN(P_A_/P_N_)) to avoid mathematical overestimation of injuries in the lower range of the function.

### Statistical analysis

Biological data were analyzed using SPSS 27 (IBM). To evaluate the significance of differences in injury rates between anesthetized and non-anesthetized fish Fisher’s exact test and Chi-square test were employed using equal sample sizes (n = 70) with a power of 80% with the possibility of detecting differences between 0.9:0.7 = 0.1:0.3 in mortality. In addition, a binary logistic regression using the anaesthetization treatment as categorical variable and the nadir pressure as continuous variable was performed. To calculate the “mortal injury metric”^76^, the contingency tables and risk function was used to calculate Odds-ratios (> 1) und Fisher’s exact Tests (P < 0.05). To determine the significance of each injury for predicting death or abnormal behavior stepwise logistic regression (Forward: LR) subsequently applied. To model the probability of injuries based on the logarithmic pressure ratios (LN(Pressure at Acclimation depth P_A_/Pressure at Nadir P_N_)) for the creation of dose-response curves, logistic regressions were performed using RegressItTM Excel Plugin and Minitab^®^software.

## Supplementary Information

Below is the link to the electronic supplementary material.

Supplementary Material 1

## Data Availability

The experimental data and the simulation results that support the findings of this study are available upon request from the corresponding author Andreas Zitek, as the project was a company co-financed project, where the contract foresees only the internal distribution of data to the project funders, while the results should be made publicly made available.

## Declarations

### Arrive guideline declaration

The methods are reported in the respective sections according to the ARRIVE guidelines (https://arriveguidelines.org).
