# Supplementary material for: Sensitivity of larval and juvenile fish with different swim bladder morphology to barotrauma with a special focus on Cypriniformes
Source: Sci Rep. 2026 Jan 7;16:2798. doi: 10.1038/s41598-025-32670-y (PMC12824194; doi:10.1038/s41598-025-32670-y)
Supplement: Supplementary file 1 — Supplementary Material 1 [file 41598_2025_32670_MOESM1_ESM.pdf]

SUPPLEMENTARY INFORMATION

Sensitivity of larval and juvenile fish with different swim bladder morphology to barotrauma with a special focus on Cypriniformes

Andreas Zitek[1,*], Wolfgang Gessl[2], Peter Mehlmauer[3], Clemens Ratschan[4], Martin Schletterer[5,6] and Josef Schneider[7]

[1]EcoScience, Vienna, 1140, Austria
[2]University of Graz, Institute of Biology, Graz, 8010, Austria
[3]Self-employed, Graz, 8020, Austria
[4]Consulting Office for Applied Hydrobiology, Fisheries and Hydro-Engineering, TB Zauner GmbH, Engelhartszell,
4090, Austria
[5] TIWAG -Tiroler Wasserkraft AG, Department of Hydropower Engineering, Innsbruck, 6020, Austria
[6] BOKU University, Institute of Hydrobiology and Aquatic Ecosystem Management, Department of Ecosystem Management, Climate and Biodiversity, Vienna, 1180, Austria
[7]Graz University of Technology, Institute of Hydraulic Engineering and Water Resources Management, Graz, 8010, Austria
[*]andreas.zitek@ecoscience.at

Species /Life stage	Anaesthetized % M	Nadir, RPC	Not Anaesthetized % M	Nadir, RPC	Statistics Fisher's exact test
E. perch L1/L2	41	18, 5.62	41	21, 4.82	*P*=0.568, *n.s.*
E. perch L4	70	19, 5.33	79	16, 6.33	*P*=0.167, *n.s*
	16	51, 1.98	13	53, 1.91	*P*=0.378, *n.s.*
	11*	control	6*	control	*P*=0.183, *n.s.*
E. perch 0+	63	15, 6.75	59	16, 6.33	*P*=0.365, *n.s.*
	4	48, 2.11	3	47, 2.16	*P*=0.500, *n.s.*
	0	control	0	control	*P=1, n.s.*
E. grayling L1/L2	0	17, 5.96	3	19, 5.33	*P=0.248, n.s.*
	1.5	50, 2.03	0	65, 1.56	*P=0.500, n.s.*
	0	control	0	control	*P=1, n.s.*
E. grayling 0+	4	17, 5.96	1.4	17, 5.96	*P*=0.500, *n.s.*
	0	55, 1.84	0	71, 1.43	*P=1, n.s.*
	0	control	0	control	*P=1, n.s.*
Roach 0+	45	17, 5.96	54	19, 5.33	*P*=0.247, *n.s*
	0	51, 1.99	0	65, 1.56	*P=1, n.s.*
	0	control	0	control	$P_{einseitig}$*=1, n.s.*

*mortalities caused by methodological issues related to the net cage.

Supplementary Table 1: Percentages of mortally injured individuals from pre-experiments in anesthetized and not anesthetized fish (n=70 per experiment) at target nadirs 15 kPa and 50 kPa and control without pressure treatment, with experimentally achieved nadirs and ratios of pressure changes (RPC) and statistical significance according to Fisher’s exact test; note that E. perch at developmental stage L4 were only available for pre-experiments.

	RoR-HPP 1, Drava		RoR-HPP 2, Mur	
Nadir	FL	PL	FL	PL
[kPa]	[%]	[%]	[%]	[%]
101	49.72	8.62	98.92	88.03
80	31.94	5.36	74.23	36.03
60	18.55	2.69	32.61	5.64
50	12.72	1.44	16.78	1.49
40	8.01	0.49	6.54	1.23
30	4.37	0.04	2.51	0.93
15	1.17	0.02	1.02	0.84

Supplementary Table 2: Summary of lowest nadirs in percent modelled by CFD along n=5000 particle traces at full load (FL) and partial load (PL).

Species	Stage	Tested concentrations mg l^{-1}	Final concentration mg l^{-1}	Description
E. grayling	L1/L2	30	30 mg/l	no abnormalities
	0+	50	50 mg/l	no abnormalities
Nase	L1	20	20 mg/l	no abnormalities
	0+	50, 30, 40, 45, 40	40 mg/l	different depths of anesthesia, depending on the different sizes of the fish.
E. perch	L1/L2	30, 20, 5	5 mg/l	fish still mobile, but at 30 or 20 mg/L, all dead after 11 minutes (3 + 8 minutes).
	L4	50, 25, 40, 35, 30	30 mg/l	no abnormalities
	L6/J1	50, 40	40 mg/l	no abnormalities
	0+	90	90 mg/l	no abnormalities
Roach	L6/J1	50	50 mg/l	no abnormalities
	0+	75, 65	65 mg/l	no abnormalities

Supplementary Table 3: Determination of the MS222 concentration needed for anesthetizing the different life stages of the investigated species as determined by two replicates with n=5.

Species / Life stage	Pre-experiments	Atmospheric pressure trials	Acclimatization trials
E. grayling L1/L2	2*70 fish at target nadir 15<br>2*70 fish at target nadir 50<br>2*70 fish for control	3*10 fish at target nadir 15<br>3*10 fish at target nadir 30<br>3*10 fish at target nadir 40<br>4*10 fish at target nadir 60<br>3*10 fish for control	-
Nase L1	-	4*10 fish at target nadir 15<br>3*10 fish at target nadir 30<br>3*10 fish at target nadir 40<br>3*10 fish at target nadir 60<br>3*10 fish for control	-
E. perch L1/L2	2*70 fish at target nadir 15	3*10 fish at target nadir 15<br>3*10 fish at target nadir 30<br>3*10 fish at target nadir 40<br>3*10 fish at target nadir 60<br>3*10 fish for control	-
E. perch L4	2*70 fish at target nadir 15<br>2*70 fish at target nadir 50<br>1*68 fish at target nadir 50<br>2*70 fish for control	-	-
E. perch L6/J1	-	3*10 fish at target nadir 15<br>3*10 fish at target nadir 30<br>3*10 fish at target nadir 40<br>3*10 fish at target nadir 60<br>3*10 fish for control	-
Roach L6/J1	-	3*10 fish at target nadir 15<br>3*10 fish at target nadir 30<br>2*10 fish at target nadir 40<br>None at target nadir 60<br>2*10 fish for control	-
Roach 0+	2*70 fish at target nadir 15<br>2*70 fish at target nadir 50<br>2*70 fish for control	3*10 fish at target nadir 15<br>3*10 fish at target nadir 30<br>3*10 fish at target nadir 40<br>3*10 fish at target nadir 60<br>3*10 fish for control	1*70 slow from 251 kPa to 101.3 kPa<br>1*70 fast from 251 kPa to 44 kPa
Nase 0+	-	3*10 fish at target nadir 15<br>3*10 fish at target nadir 30<br>3*10 fish at target nadir 40<br>3*10 fish at target nadir 60<br>3*10 fish for control	1*70 slow from 251 kPa to 101.3 kPa<br>1*70 fast from 251 kPa to 29 kPa
E. grayling 0+	2*70 fish at target nadir 15<br>2*70 fish at target nadir 50<br>2*70 fish for control	3*10 fish at target nadir 15<br>3*10 fish at target nadir 30<br>3*10 fish at target nadir 40<br>3*10 fish at target nadir 60<br>3*10 fish for control	1*70 slow from 251 kPa to 101.3 kPa<br>1*70 fast from 251 kPa to 44 kPa<br>1*70 fast from 251 kPa to 42 kPa*
E. perch 0+	2*70 fish at target nadir 15<br>2*70 fish at target nadir 50<br>2*70 fish for control	3*10 fish at target nadir 15<br>3*10 fish at target nadir 30<br>3*10 fish at target nadir 40<br>3*10 fish at target nadir 60<br>3*10 fish for control	1*70 slow from 251 kPa to 101.3 kPa<br>1*70 fast from 251 kPa to 15 kPa

Supplementary Table 4: Overview on the number of fish used for different types of experiments; all experiments were run with anaesthetized fish, except for one experiment of acclimatized E. grayling 0+ (*), where the experiment was repeated without anesthetization to exclude a potential effect of the anaesthetic on the unexpected results.

Parameter	RoR-HPP 1, river Drava (MAF = 195 m^3 s^{-1})	RoR-HPP 2, river Mur (MAF = 117 m^3 s^{-1})
Turbine Type	Kaplan 5100 (n=2), vertical	Kaplan RT 3850 (n=2), horizontal with 10° inclination
Maximum Flow Rate per Turbine (m^3 s^{-1})	275	104
Average Turbine Flow Rate (m^3 s^{-1})	230	58
Number of Blades	5	4
Flow Rate at Best Efficiency (m^3 s^{-1})	170	71
Turbine Runner Diameter (mm)	5100	3850
Maximum Turbine Efficiency (%)	94.6	88.7
RPM (Rotations per Minute)	142.9	125
Head Difference (Upper-Lower Water) (m)	25.2	7.36
Reservoir Depth (m)	25–30	7
Clear Width of Trash Rack (mm)	140	100

Supplementary Table 5: Parameters of two studied Kaplan turbines typical for Run-Of-River Hydropower Plants (RoR-HPP) at medium sized rivers (MAF = mean annual flow).

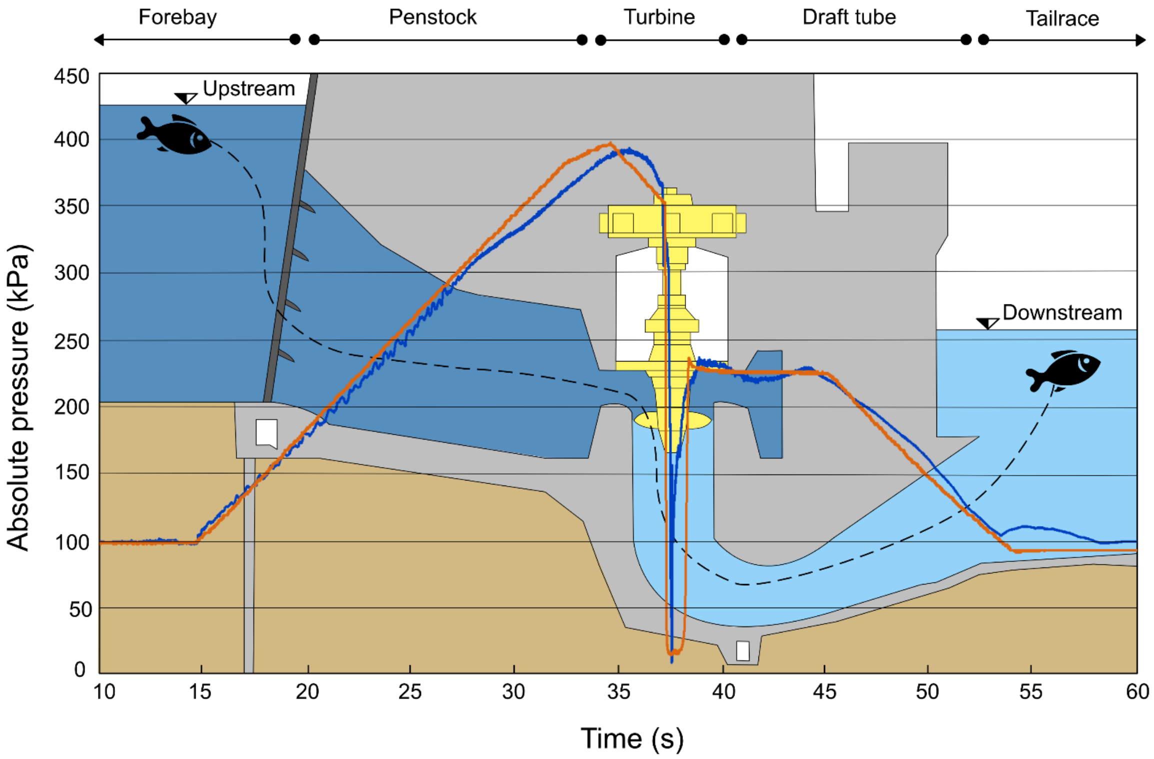

Supplementary Figure 1: Section trough a typical Run-Of-River Hydropower-Plant (RoR-HPP) with a vertical Kaplan turbine, and example of a pressure curve experienced by a fish passing through (dotted black line), a sensor-measured pressure curve (blue line) and a pressure curve simulated by the barotrauma chamber (orange line)[1].

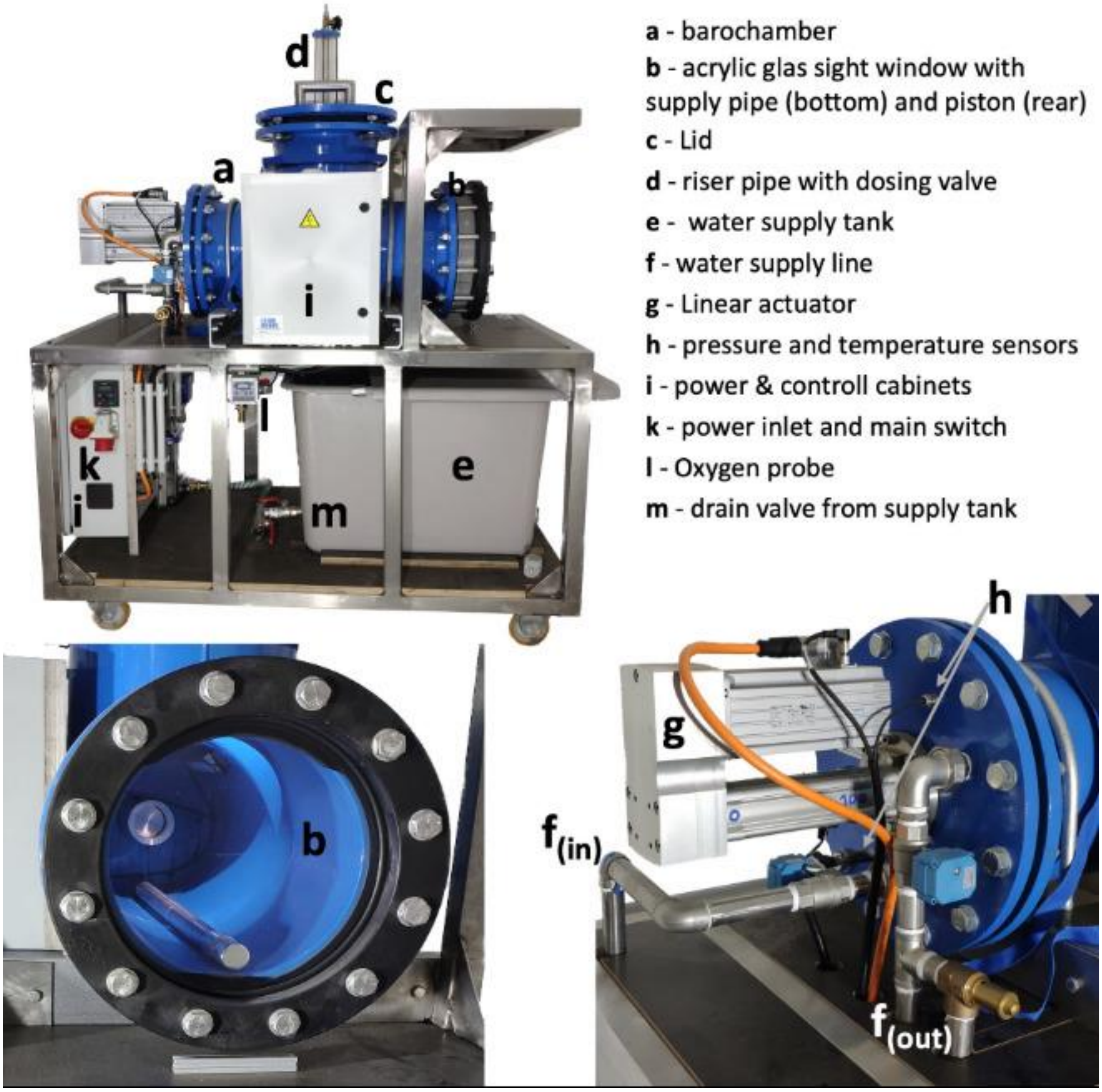

Supplementary Figure 2: Main components of the barotrauma chamber[1].

References

1 Schneider, J., Haas, C., Thumser, P., Hauna, S., Schmid, G., Noack, M., Ratschan, C., Schletterer, M. & Zitek, A. A barotrauma chamber for the systematic assessment of barotrauma-related injuries in potamodromous European fish species. *J. Ecohydraulics*, 1–11, https://doi.org/10.1080/24705357.2025.2541435 (2025).
